# Murine Myoblasts Exposed to SYUIQ-5 Acquire Senescence Phenotype and Differentiate into Sarcopenic-Like Myotubes, an In Vitro Study

**DOI:** 10.1093/gerona/glae022

**Published:** 2024-01-24

**Authors:** Laura Gerosa, Amir Mohammad Malvandi, Marta Gomarasca, Chiara Verdelli, Veronica Sansoni, Martina Faraldi, Ewa Ziemann, Fabiola Olivieri, Giuseppe Banfi, Giovanni Lombardi

**Affiliations:** Laboratory of Experimental Biochemistry and Molecular Biology, IRCCS Istituto Ortopedico Galeazzi, Milano, Italy; Laboratory of Experimental Biochemistry and Molecular Biology, IRCCS Istituto Ortopedico Galeazzi, Milano, Italy; Laboratory of Experimental Biochemistry and Molecular Biology, IRCCS Istituto Ortopedico Galeazzi, Milano, Italy; Laboratory of Experimental Biochemistry and Molecular Biology, IRCCS Istituto Ortopedico Galeazzi, Milano, Italy; Laboratory of Experimental Biochemistry and Molecular Biology, IRCCS Istituto Ortopedico Galeazzi, Milano, Italy; Laboratory of Experimental Biochemistry and Molecular Biology, IRCCS Istituto Ortopedico Galeazzi, Milano, Italy; Department of Athletics, Strength and Conditioning, Poznań University of Physical Education, Poznań, Poland; Clinic of Laboratory and Precision Medicine, IRCCS INRCA, Ancona, Italy; Department of Clinical and Molecular Sciences, Università Politecnica delle Marche, Ancona, Italy; Laboratory of Experimental Biochemistry and Molecular Biology, IRCCS Istituto Ortopedico Galeazzi, Milano, Italy; Faculty of Medicine and Surgery, Vita-Salute San Raffaele University, Milan, Italy; Laboratory of Experimental Biochemistry and Molecular Biology, IRCCS Istituto Ortopedico Galeazzi, Milano, Italy; Department of Athletics, Strength and Conditioning, Poznań University of Physical Education, Poznań, Poland; (Biological Sciences Section)

**Keywords:** In vitro model, Sarcopenic myotubes, Senescence, Senescent myoblasts, SYUIQ-5

## Abstract

The musculoskeletal system is one of the most affected organs by aging that correlates well with an accumulation of senescent cells as for other multiple age-related pathologies. The molecular mechanisms underpinning muscle impairment because of senescent cells are still elusive. The availability of in vitro model of skeletal muscle senescence is limited and restricted to a small panel of phenotypic features of these senescent cells in vivo. Here, we developed a new in vitro model of senescent C2C12 mouse myoblasts that, when subjected to differentiation, the resulting myotubes showed sarcopenic features. To induce senescence, we used SYUIQ-5, a quindoline derivative molecule inhibitor of telomerase activity, leading to the expression of several senescent hallmarks in treated myoblasts. They had increased levels of p21 protein accordingly with the observed cell cycle arrest. Furthermore, they had enhanced SA-βgalactosidase enzyme activity and phosphorylation of p53 and histone H2AX. SYUIQ-5 senescent myoblasts had impaired differentiation potential and the resulting myotubes showed increased levels of ATROGIN-1 and MURF1, ubiquitin ligases components responsible for protein degradation, and decreased mitochondria content, typical features of sarcopenic muscles. Myotubes differentiated from senescent myoblasts cultures release increased levels of MYOSTATIN that could affect skeletal muscle cell growth. Overall, our data suggest that a greater burden of senescent muscle cells could contribute to sarcopenia. This study presents a well-defined in vitro model of muscle cell senescence useful for deeper investigation in the aging research field to discover new putative therapeutic targets and senescence biomarkers associated with the aged musculoskeletal system.

## Graphical Abstract

**Graphical Abstract:**
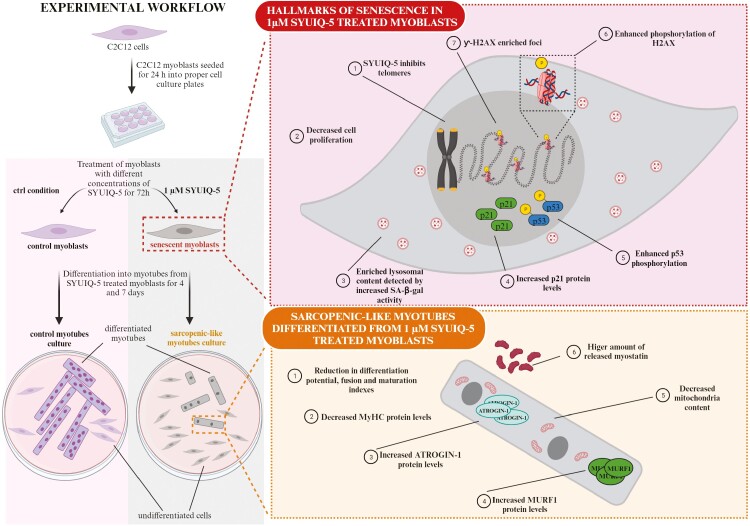

Aging is a physiological process that involves several pathways concurring to various organ dysfunction and age-related diseases. The musculoskeletal apparatus is one of the most affected organs impaired during aging. More than 50% of the population over 80 years (1) manifests loss of muscle mass and strength, a muscle-wasting condition, known as sarcopenia, that is, often associated with bone frailty loss leading to enhanced fracture risk and disability (2). Because age-associated muscle wasting affects a very large proportion of the population, the implementation of strategies that prevent muscle loss becomes of high clinical relevance. However, despite the improvement in the knowledge about the pathophysiological alterations observed in muscle aging, the specific mechanisms underpinning the musculoskeletal decline typical of older people are not well known.

Accumulation of senescent cells during aging contributes to diffuse organ dysfunctions (3). In particular, studies performed on murine models of aging have demonstrated that the presence of senescent cells could have detrimental effects also on skeletal muscle functions (4). However, a specific link between the accumulation of senescent cells and muscle impairment needs more investigation. Cellular senescence is a physiological, protective process characterized by an irreversible cell cycle arrest (5). Despite that, senescent cells acquire a pro-inflammatory senescence-associated secretory phenotype (SASP), that alters the microenvironment and leads to detrimental changes in neighboring cells spreading senescence in a paracrine manner (6). Thus, the accumulation of senescent cells during aging affects the correct tissue functionality. Several stressor stimuli could induce senescence and, depending on cell type and type and timing of the stimuli, senescence leads to the expression of a heterogeneous phenotype. Among the several phenotypic features characterizing senescent cells, the most common is the accumulation of lysosomal content detectable by increased of β-galactosidase enzymatic activity, reduced telomere length, DNA damage response (DDR) activation, cell cycle arrest, and organelle impairment, that is, mitochondrial dysfunction (3,7). Thus, the assessment of senescent cells is based on identifying some of these features together (7).

A few studies have investigated the correlation between the senescent state in muscles and the musculoskeletal impairment typical of aged people. Different cell types, such as satellite cells, myocytes, fibroblasts, nerve fibers, and endothelial cells, compose the skeletal muscle tissue. Studies on murine models demonstrated that some of these cells could be susceptible to cellular senescence with chronological aging (8,9). In vitro studies are useful to investigate the molecular mechanisms of muscle dysfunctions due to senescent cell accumulation. However, known in vitro models of senescent muscle cells are few and suffer from important limitations.

With this study, we aimed at obtaining an in vitro model of senescent myoblasts by treating C2C12 cells with an inhibitor of telomerase activity, SYUIQ-5 (10). Here, we describe the procedures applied and the results obtained from the characterization of this in vitro model of senescent murine skeletal muscle cells.

## Method

### Cell Culture and SYUIQ-5 Treatment

Mouse skeletal myoblasts C2C12 (ATCC, Manassas, VA, USA) were cultured in growth medium, composed of high-glucose Dulbecco’s modified Eagle medium (DMEM; Thermo Fisher Scientific, Waltham, MA) supplemented with 10% HyClone fetal bovine serum (Cytiva, Marlborough, MA, USA), 1·10^5^ U/L penicillin, 0.1 mg/L streptomycin, and 2 mM L-glutamine (all from Thermo Fisher Scientific). Cells were kept at 37° C in a 5% CO_2_ humidified atmosphere.

Different concentrations of SYUIQ-5 (# S5826, Merck, Darmstadt, Germany) were added to the culture medium and kept for 72 hours. Then, cells were either collected for the analyses or differentiated into myotubes. Cells have been counted with NucleoCounter (Chemometec NC-3000, Allerod, Denmark) according to the manufacturer’s instructions.

C2C12 myoblasts were differentiated into myotubes by using a differentiation medium composed of high-glucose DMEM supplemented with 2% horse serum (Gibco, LifeTechnologies, Carlsbad, CA, USA), 1·10^5^ U/L penicillin, 0.1 mg/L streptomycin, and 2 mM L-glutamine, replacing it every 48 hours for 4 or 7 days.

### Cytotoxic Assay

C2C12 cells were seeded in a 96-well plate at a density of 3 000 cells/cm^2^ and treated as described above. SYUIQ-5 cytotoxicity was evaluated using a cytotoxic assay (CytoTox96 Non-Radioactive Cytotoxicity Assay, Promega, Madison, WI, USA) according to the manufacturer’s instructions. Supernatants derived from control and treated cells were collected. Cells were lysed using a lysis buffer for 45 minutes at room temperature. CytoTox96 Reagent was added in equal volume to the collected supernatant or to the cellular lysates and incubated in the dark for 30 minutes at room temperature. Stop solution was added and absorbance, at λ = 490 nm, was read using VICTOR X3 Multilabel Counter (Perkin Elmer, Inc., Waltham, MA, USA) to detect the amount of lactic acid dehydrogenase (LDH) in supernatant and cell lysate.

### Cell Viability

C2C12 cells were seeded in a 96-well plate at a density of 3 000 cells/cm^2^ and treated with 0 µM, 0.4 µM, 0.7 µM, 1 µM, and 2 µM of SYUIQ-5 for 2 hours (acute treatment) and 72 hours (chronic treatment), at 37° C. Cell viability was indirectly assayed by measuring the mitochondrial reducing potential with the AlamarBlue assay. 10× AlamarBlue reagent was added to cells and incubated for 4 hours; fluorescence emission was measured at λ = 585 nm, following excitation at λ = 570 nm, by VICTOR X3 Multilabel Counter (Perkin Elmer, Inc). Protein content was determined by Pierce BCA protein assay kit (Thermo Fisher Scientific).

### Cellular Proliferation Assay

C2C12 cells were seeded in a 96-well plate at a density of 3 000 cells/cm^2^ and treated with 0 µM, 0.4 µM, and 1.0 µM of SYUIQ-5 for 72 hours. Cell proliferation was assayed by a colorimetric ELISA-BrdU kit (Roche Diagnostics, Basel, Switzerland). Briefly, after SYUIQ-5 treatments, cells were incubated with BrdU labeling solution for 6 hours at 37° C. Labeled cells were fixed and incubated with antibody-conjugated anti-BrdU-POD for 90 minutes at room temperature. After washing, substrate solution was added for 10 min at room temperature. Finally, BrdU incorporation into the DNA of proliferating cells was measured by VICTOR X3 Multilabel Counter reading absorbance at λ = 450 nm (reference wavelength λ = 690 nm; Perkin Elmer, Inc.).

### 9H-(1,3-dichloro-9,9-dimethylacridin-2-one-7-yl) beta-d-galactopyranoside (DDAOG) Staining and FACS Analysis for SA-β-galactosidase Activity Detection

In the presence of β-galactosidase, DDAOG substrate (#D6488, Thermo Fisher Scientific) is hydrolyzed to 7-hydroxy-9H(I,3-dichloro-9,9-dimethylacridin-2-one) (DDAO), generating a far-red fluorescent signal. After induction of senescence in C2C12 cells with SYUIQ-5 (NT, 0.4 µM and 1.0 µM for 72 hours), in accordance with previously published papers, but with some modifications (11), cells were pretreated 1 hour before being harvested with 100 nM bafilomycin-A1 (Merck) to induce lysosome alkalinization, detached by TryP1E (Gibco, LifeTechnologies) and stained for viability with live/dead fixable near-IR dead cell stain kit (Thermo Fisher Scientific) for 30 minutes, at room temperature according to manufacturer’s instructions. Cells were washed once in PBS and fixed for 15 minutes with paraformaldehyde 2% in PBS/FBS 1% at room temperature. After being washed, cells were permeabilized with 0.2% saponin in PBS/FBS 1% and then incubated with 0.02 mM DDAOG in proper buffer (PBS/FBS 1%, MES hydrate, 0.2% saponin) for 2 hours at room temperature. Finally, cells were analyzed on a flow cytometer (CytoFlex, Beckman Coulter, Brea, CA, USA).

### RNA Isolation and Real-Time Quantitative Reverse Transcription (RT-qPCR)

Total RNA was isolated from SYUIQ-5-treated cells (NT, 0.4 µM and 1 µM for 72 hours, 37° C) using TRIzol reagent (Invitrogen, Thermo Fisher Scientific) following the manufacturer’s instructions. RNA concentration was determined using NanoDrop spectrophotometer (Thermo Fisher Scientific) and DNA contamination was removed by DNaseI (Invitrogen) treatment. Reverse transcription reaction was carried out using the iScript cDNA Synthesis Kit (Bio-Rad Laboratories, Hercules, CA, USA) and RT-qPCR was performed on QuantStudio 12K Flex Real-Time PCR System (Applied Biosystem, Thermo Fisher Scientific) using the following Taqman gene expression assays: p21 (Cdkn1a, Mm00432448_m1). As potential reference genes, GAPDH (Mm99999915_g1), TBP (Mm01277042_m1), and RPL0 (Mm00725448_s1) were tested using the NormFinder algorithms and GAPDH resulted in the most stable one. The relative expression of p21 was calculated by the 2^−ΔΔCq^ method, using GAPDH as a reference gene.

### Protein Extraction and Immunoblotting

Proteins were extracted from SYUIQ-5 treated myoblasts (NT, 0.4 µM and 1 µM for 72 hours, 37° C) and from myotubes differentiated for 7 days from SYUIQ-5-treated myoblasts using NP40 lysis buffer (Thermo Fisher Scientific) containing proteases and phosphatases inhibitors. Total protein concentration was determined by BCA assay (Thermo Fisher Scientific) and 15 µg or 20 µg of cell lysates were resolved into a 12% or 14% SDS-polyacrylamide gel and transferred to 0.2 µM pore-size PVDF (Bio-Rad Laboratories), according to standard procedures. For immunoblotting the following primary antibodies were used: p21 (#AMab109199, Prodotti Gianni s.r.l., Milan, Italy); phosphorylated H2AX (Ser139) and H2AX (#2577 and #2595, respectively; Cell Signaling Technologies, Danvers, MA, USA); phosphorylated p53 (Ser15) and p53 (#9286 and #9293, respectively; Cell Signaling Technologies); LC3A/B (#4108, Cell Signaling Technologies); CASPASE-7 (#12827, Cell Signaling Technologies); CASPASE-3 (#14220, Cell Signaling Technologies); myosin heavy chain (MyHC, #MAB4470, Bio-Techne, Minneapolis, MI, USA); MURF1 (#AF5366, Bio-techne); ATROGIN-1 (#Ab168372, Abcam, Cambridge, UK and #AM3141, ECM Biosciences, Versailles, KY, USA); TOTAL UBIQUITIN (#, Proteintech Genomics, San Diego, CA, USA). Anti-VINCULIN antibody (#ab129002, Abcam) and anti-β-ACTIN (#TA811000, Origene, Rockville, MD, USA) were used as internal control. Appropriate secondary antibodies, conjugated to horseradish peroxidase (Santa Cruz Biotechnology, Dallas, TX, USA), were used and specific bands were detected with ChemiDoc Imaging System. Acquired images were analyzed through Image Lab software (Bio-Rad Laboratories) and protein expression levels were normalized using VINCULIN as reference.

### Immunofluorescence and Image Acquisition and Analysis

Myoblasts treated with 5 µM etoposide (#341205, Merck) for 1 hour, as positive control for H2AX nuclear foci enrichment, or with SYUIQ-5 for 72 hours, and myotubes differentiated for 4 and 7 days from SYUIQ-5-treated myoblasts were fixed with 4% paraformaldehyde (Alfa Aesar, Thermo Fisher Scientific) for 10 minutes at room temperature. Cells were permeabilized with 0.1% TritonX100 (Merck) for 10 minutes at room temperature and blocked with blocking solution (0.1% saponin and 5% BSA in PBS buffer) for 45 minutes at room temperature. Cells were then incubated with primary (overnight, at 4° C) and secondary (1 hour at room temperature) antibodies diluted in antibody solution (blocking solution diluted 1:10 in PBS buffer). The following primary antibodies were used: anti-TUBULIN (1:400, #T4026 Merck); anti-phosphorylated H2AX (Ser139, ƴ-H2AX, 1:800, #2577 Cell Signaling Technologies); anti-MyHC (1:200, #MAB4470, Bio-Techne); anti-MURF1 (1:200, #AF5366, Bio-techne); anti-ATROGIN1 (1:100, #AM3141, ECM Biosciences). Secondary antibodies conjugated to the following dies were used: Alexa Fluor 488 (1:1000, Invitrogen); Alexa Fluor 568 (1:1000, Invitrogen); Alexa Fluor 647 (1:1000, Invitrogen). Nuclei were stained with DAPI (1:10000, Invitrogen) diluted in PBS buffer for 5 minutes at room temperature. For mitochondria staining, myotubes differentiated for 7 days were incubated with MitoTracker Red CMXRos (#M7512 Invitrogen) 100 nM for 30 minutes, at 37° C and then fixed with 4% paraformaldehyde for 10 minutes, at RT, before the previously described immunofluorescence procedure. Images were acquired using a Leica SP8 laser scanning microscope (Leica Microsystems, Wetzlar, Germany) with either a 20× or oil immersion 100× objectives. Images were obtained from the *z*-projection (maximum intensity) of almost 10 stacks taken at 0.75 µm intervals at 1024 × 1024 pixel resolution. Confocal images were analyzed with Fiji software (12). In particular, in myotubes differentiation analyses DAPI channel was used to count nuclei, and the MyHC channel was used to mark and count myotubes and differentiated myoblasts. Three parameters were used to characterize the myotubes: differentiation index, fusion index, and maturation index, according to (13). The differentiation index has been calculated as the number of MyHC-positive cells, normalized on the total number of nuclei. The fusion index has been calculated as the number of nuclei in myotubes containing 2 or more nuclei, normalized on the total number of nuclei. The maturation index has been calculated as the number of myotubes containing 5 or more nuclei, normalized on the number of myotubes containing 2 or more nuclei.

For MitoTracker analyses, MyHC channel was used to draw regions of interest and the mean fluorescence intensity of MitoTracker was quantified on z-stack projections.

### Caspase 3/7 Enzymatic Activities

Caspase 3/7 enzymatic activities were analyzed in myoblasts treated with SYUIQ-5 for 72 h (NT, 0.4 µM, 1.0 µM) or with 50 µM etoposide for 24 hours at 37° C as a positive control for apoptosis, and compared with untreated cells (NT), by using Apo-ONE Homogeneous caspase-3/7 assay (Promega), accordingly to manufacturer’s instructions. Briefly, cells were seeded into 96 multi-well plates and, after SYUIQ-5 and etoposide treatments, Apo-ONE caspase 3/7 Reagent was added to the cultures and kept for 10 hours. After incubation, the fluorescence signal was measured by VICTOR X3 Multilabel Counter (Perkin Elmer).

### Statistical Analysis

**Figure 1.**
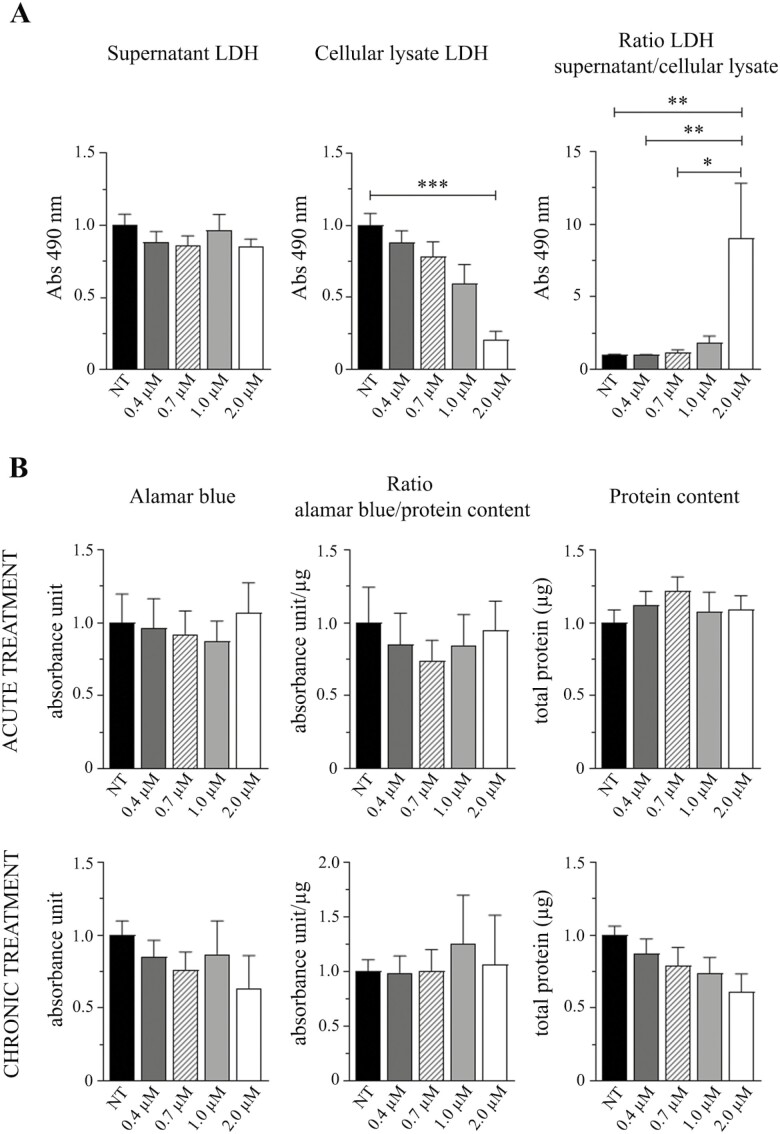
Cytotoxic effects and murine myoblasts viability analysis after SYUIQ-5 treatments. (**A**) Histograms relative to LDH enzyme detection in C2C12 cells untreated (NT) or treated (0.4 μM, 0.7 μM, 1.0 μM, and 2 μM) with SYUIQ-5 for 72 h: LDH released in the supernatant (left panel) (*n* = 8 ns, Kruskal–Wallis test, Dunn’s multiple comparison test); LDH in cell lysates (middle panel; *n* = 8, NT vs 2 μM ***p = .0003, Kruskal–Wallis test, Dunn’s multiple comparison test); LDH released in supernatant normalized on LDH present in cellular lysates (right panel; *n* = 8, NT vs 2 μM **p = .0032, 0.4 μM vs 2 μM ** *p* = .0056, 0.7 μM vs 2 μM *p = .0163, Kruskal–Wallis test, Dunn’s multiple comparison test). (**B**) Histograms relative to Alamar Blue assay (left panel) (acute treatment: *n* = 4 ns, one-way ANOVA Dunnett’s multiple comparison test; chronic treatment: *n* = 4 ns, 1-way ANOVA Dunnett’s multiple comparison test). Histograms relative to Alamar Blue detection signal normalized on protein content (middle panel; acute treatment: *n* = 4 ns, 1-way ANOVA Dunnett’s multiple comparison test; chronic treatment: *n* = 4 ns, 1-way ANOVA Dunnett’s multiple comparison test). Histograms relative to protein content (right panel) (acute treatment: *n* = 4 ns, 1-way ANOVA Dunnett’s multiple comparison test; chronic treatment: *n* = 4 ns, 1-way ANOVA Dunnett’s multiple comparison test). Cells have been untreated (NT) or treated with SYUIQ-5 (0.4 μM, 0.7 μM, 1.0 μM, and 2.0 μM) for 2 h (acute treatment) or for 72 h (chronic treatment).

**Figure 2.**
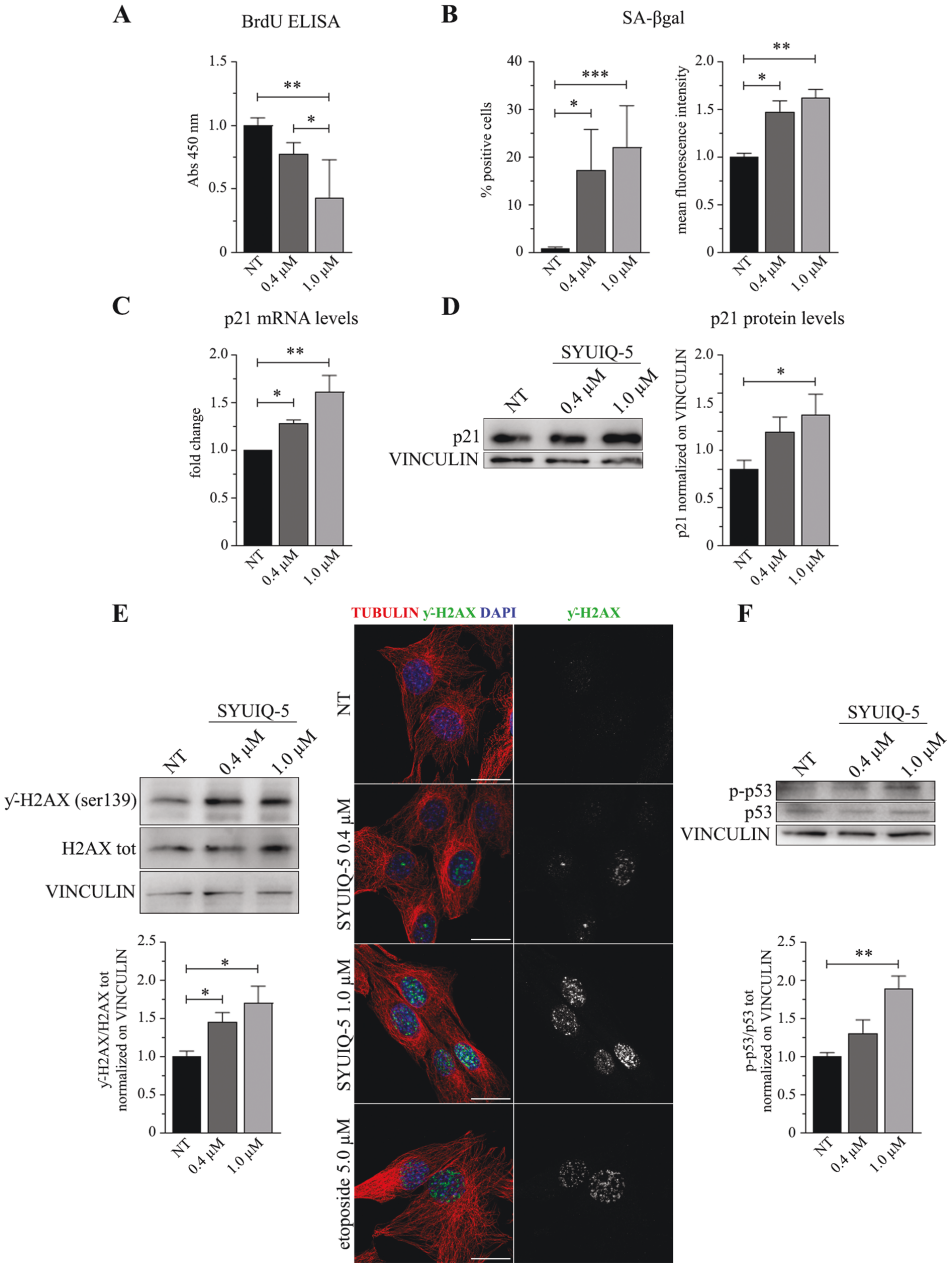
Hallmarks of senescence induced by SYUIQ-5 in murine myoblasts. C2C12 cells have been untreated (NT) or treated with SYUIQ-5 (0.4 μM and 1.0 μM) for 72 h and then analyzed for several senescent markers as shown in the following results: (**A**) histogram relative to BrdU ELISA (*n* = 5, NT vs 1.0 μM ***p = .0010, 0.4 μM vs 1.0 μM *p = .0282, 1-way ANOVA Tukey’s multiple comparison test). (**B**) FACS analysis results performed on SYUIQ-5 untreated (NT) or treated (0.4 μM and 1.0 μM) myoblasts pre-incubated with DDAOG as a fluorimetric substrate for SA-βgal enzyme and detected at 660 nm. Percentage of cells expressing SA-βgal (*n* = 8, NT vs 0.4 μM *p = .0174, NT vs 1.0 μM ***p = .0002, Kruskal–Wallis Dunn’s multiple comparison test); mean intensity fluorescence signal of DDAO converted from DDAOG substrate by SA-βgal enzyme (*n* = 8, NT vs 0.4 μM *p = .0267, NT vs 1.0 μM **p = .0012, Kruskal–Wallis Dunn’s multiple comparison test). (**C**) Relative mRNA levels of p21 transcript shown as fold change (*n* = 6, NT vs 0.4 μM *p = .0396, NT vs 1.0 μM **p = .0016, Kruskal–Wallis Dunn’s multiple comparison test). (**D**) Western blot analysis of cellular lysates of SYUIQ-5 treated myoblasts and quantification of p21 protein levels normalized on VINCULIN protein (*n* = 9, NT vs 1.0 μM *p = .0409, Kruskal–Wallis Dunn’s multiple comparison test). (**E**) Western blot analysis (left panel) of cellular lysates of SYUIQ-5 treated myoblasts and quantification of phosphorylated histone H2AX (ƴ-H2AX) compared to total histone H2AX and normalized on VINCULIN (*n* = 5, NT vs 0.4 μM *p = .0467, NT vs 1.0 μM *p = .0173, Kruskal–Wallis Dunn’s multiple comparison test). Representative confocal images of ƴ-H2AX foci enrichment in nucleus of myoblasts treated with SYUIQ-5 or with etoposide as positive control. Objective oil immersion 100×. Scale bar 10 μm. (**F**) Western blot analysis of cellular lysates of SYUIQ-5 treated myoblasts and quantification of phosphorylated p53 (p-p53) compared to total p53 and normalized on VINCULIN (*n* = 5, NT vs 1.0 μM **p = .0056, Kruskal–Wallis Dunn’s multiple comparison test).

**Figure 3.**
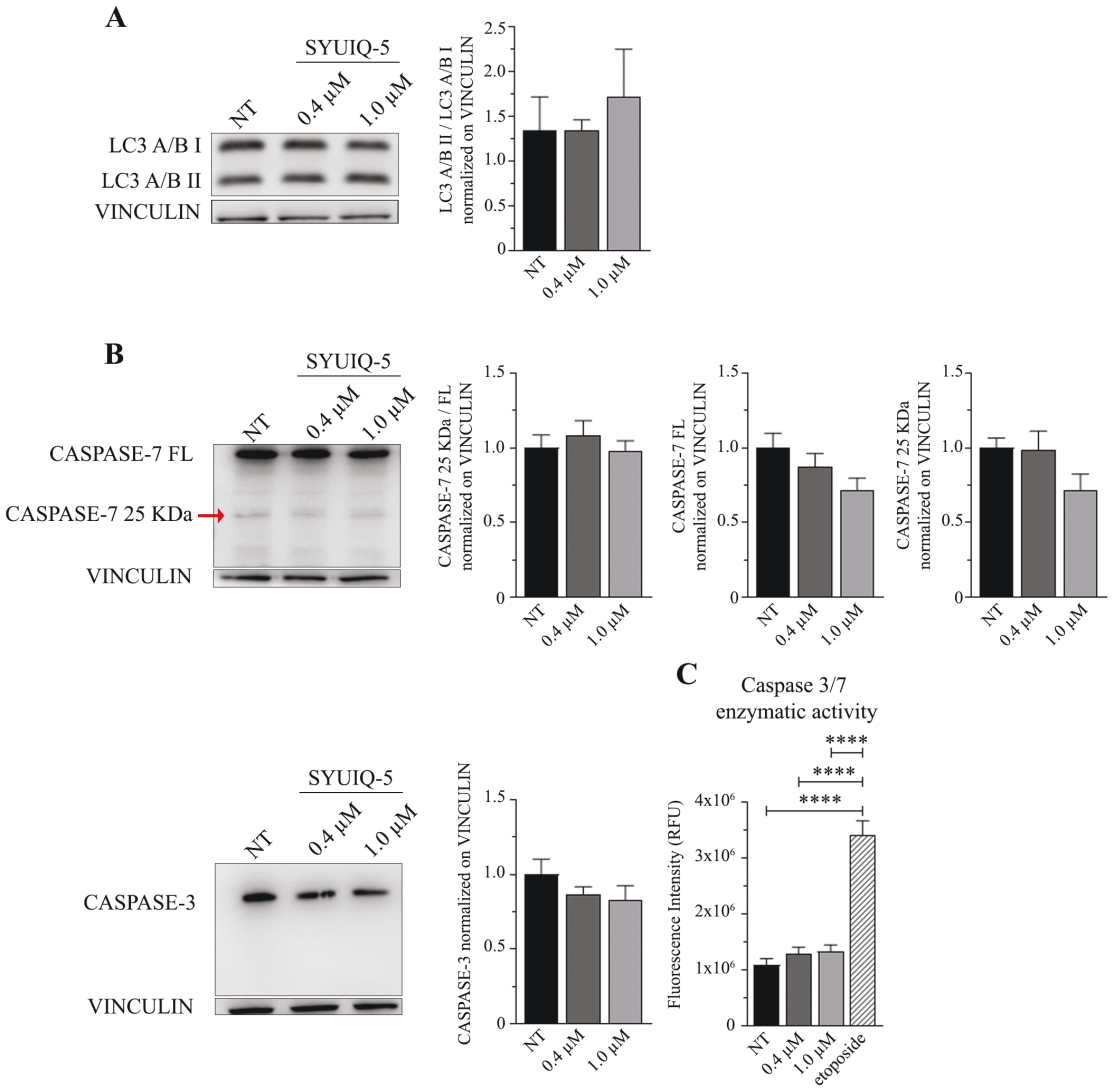
SYUIQ-5 did not activate autophagy nor apoptotic pathways in C2C12 cells. C2C12 cells have been untreated (NT) or treated with SYUIQ-5 (0.4 μM and 1.0 μM) for 72 h and lysates for the following western blot analysis: (**A**) representative western blot images of LC3A/B signals (LC3A/B I upper band and LC3A/B II down band) and quantification of LC3A/B II cleaved fragment compared to LC3A/B I normalized on VINCULIN (*n* = 3 ns, Kruskal–Wallis Dunn’s multiple comparison test); (**B**) representative western blot images of CASPASE-7 signal (CASPASE full-length FL, 35 KDa, CASPASE-7 cleaved fragment 25 KDa indicated by arrow). Histograms relative to: quantification of cleaved CASPASE-7 compared to FL form and normalized on VINCULIN (left panel, *n* = 7 ns, Kruskal–Wallis Dunn’s multiple comparison test); quantification of CASPASE-7 FL normalized on VINCULIN (middle panel, *n* = 7 ns, Kruskal–Wallis Dunn’s multiple comparison test); quantification of cleaved CASPASE-7 fragment normalized on CASPASE-7 FL (right panel, *n* = 7 ns, Kruskal–Wallis Dunn’s multiple comparison test). Representative western blot images of CASPASE-3 signal and quantification of CASPASE-3 normalized on VINCULIN (*n* = 8 ns, Kruskal–Wallis Dunn’s multiple comparison test). (**C**) Fluorescence intensity detection of caspase 3/7 enzymatic activity on myoblasts untreated (NT) or treated with SYUIQ-5 (0.4 μM and 1.0 μM) or with etoposide as the positive control (*n* = 6–9, NT vs 1.0 μM ****p < .0001, Kruskal–Wallis Dunn’s multiple comparison test).

**Figure 4.**
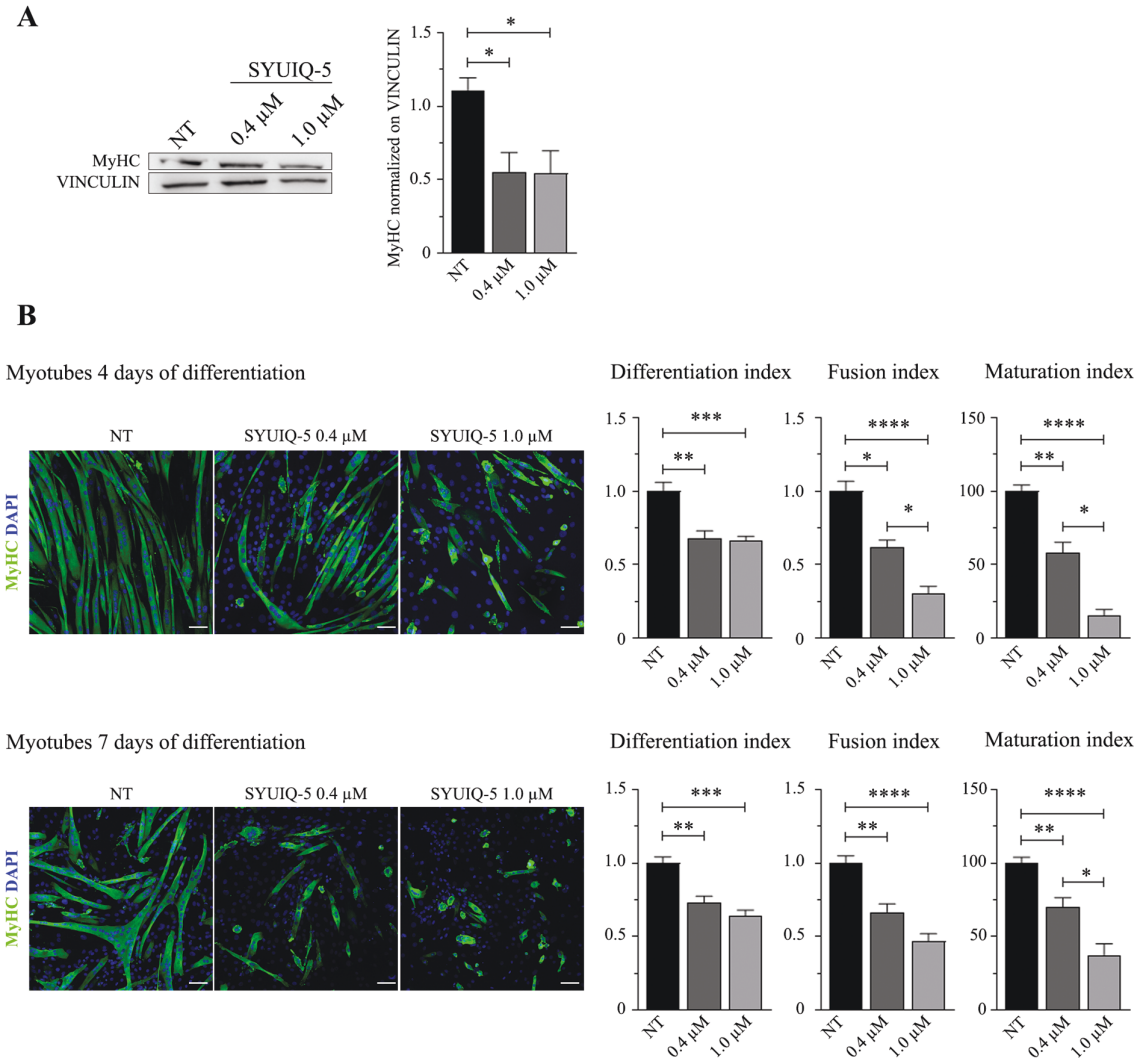
Impairment of SYUIQ-5 treated myoblasts differentiation into myotubes. (**A**) Representative western blot images of MyHC protein expressed in myotubes lysates differentiated for 7 d from SYUIQ-5 untreated (NT) or treated (0.4 μM and 1.0 μM) myoblasts. Histograms relative to quantification of MyHC protein levels normalized on VINCULIN (*n* = 6, NT vs 0.4 μM *p = .030, NT vs 1 μM *p = .0452, Kruskal–Wallis Dunn’s multiple comparison test). (**B**) Representative confocal images of myotubes differentiated for 4 d (top panel) or for 7 d (bottom panel) from SYUIQ-5 untreated (NT) or treated (0.4 μM and 1.0 μM) myoblasts. Cells have been immunolabeled with anti-MyHC (green channel) to count differentiated cells and nuclei have been stained with DAPI (blue channel) to count the number of nuclei. Objective 20×, scale bar, 20 μM. Histograms indicate differentiation index (DI); fusion index (FI); maturation index (MI). Histograms relative to 4 d myotubes: DI (*n* = 14, NT vs 0.4 μM **p = .0025, NT vs 1.0 μM ***p = .0001); FI (*n* = 14, NT vs 0.4 μM *p = .159, NT vs 1.0 μM ****p < .0001, 0.4 μM vs 1.0 μM *p = .0317); MI (*n* = 14, NT vs 0.4 μM **p = .0086, NT vs 1.0 μM ****p < .0001, 0.4 μM vs 1.0 μM *p = .0291). Histograms relative to 7 d myotubes: DI (*n* = 17–23, NT vs 0.4 μM **p = .0011, NT vs 1.0 μM ****p < .0001); FI (*n* = 17–23, NT vs 0.4 μM **p = .0019, NT vs 1.0 μM ****p < .0001); MI (*n* = 17–23, NT vs 0.4 μM **p = .0059, NT vs 1.0 μM ****p < .0001, 0.4 μM vs 1.0 μM *p = .0404). Statistical analysis: Kruskal–Wallis Dunn’s multiple comparison test.

**Figure 5.**
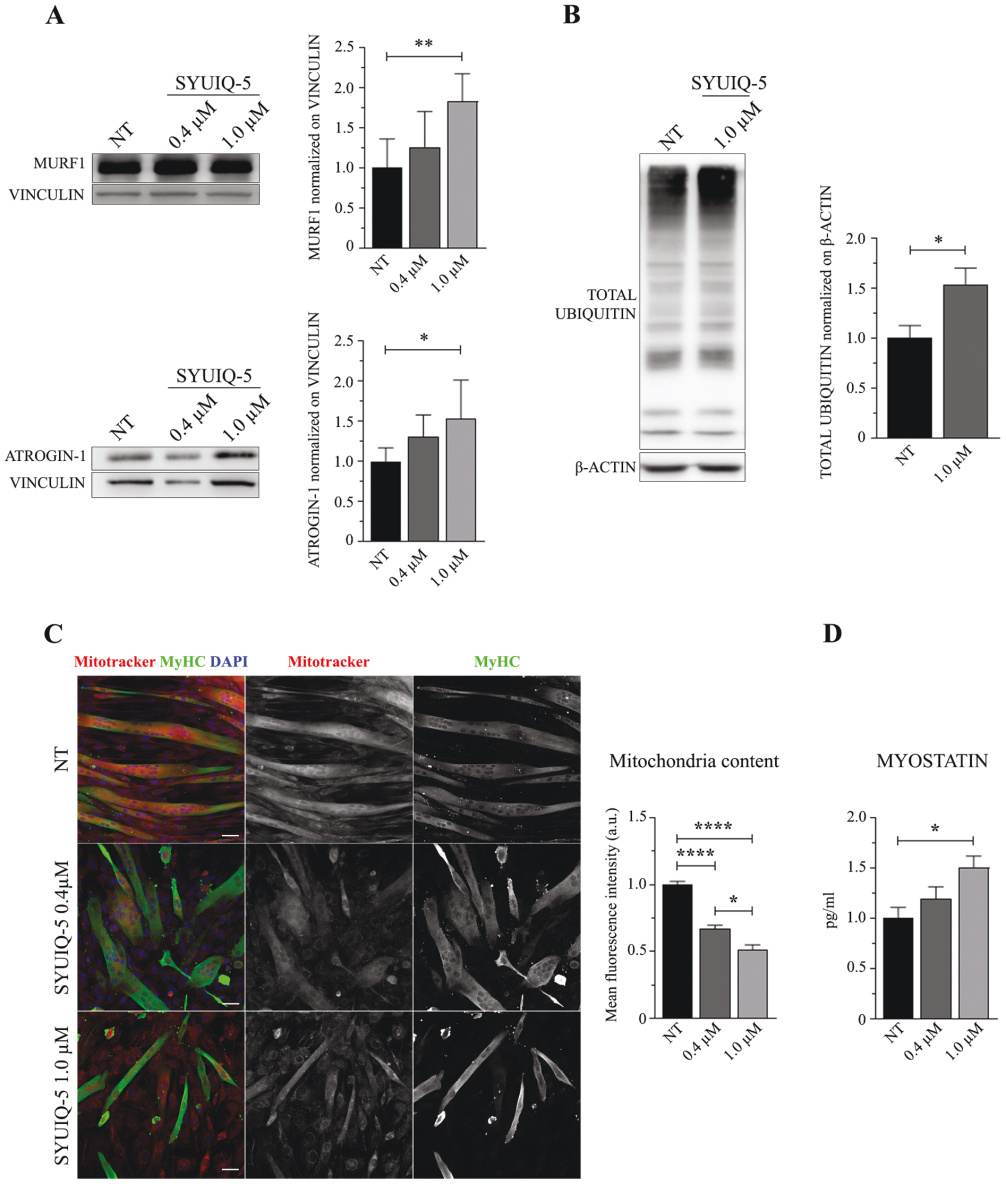
Sarcopenic features of myotubes differentiated from senescent SYUIQ-5 treated myoblasts. (**A**) Representative western blot images of MURF1 and ATROGIN-1 proteins expressed in myotubes lysates differentiated for 7 d from SYUIQ-5 untreated (NT) or treated (0.4 μM and 1.0 μM) myoblasts and relative histograms of protein levels quantification normalized on VINCULIN (MURF1 histogram: *n* = 5–7, NT vs 1.0 μM **p = .059, 1-way ANOVA Tukey’s multiple comparison test; ATROGIN-1 histogram: *n* = 6, NT vs 1.0 μM *p = .0370, 1-way ANOVA Tukey’s multiple comparison test). (**B**) Representative western blot images of TOTAL UBIQUITIN protein expressed in myotubes differentiated for 7 d from SYUIQ-5 untreated (NT) or treated (1.0 μM) myoblasts and relative histogram of protein levels quantification normalized on β-ACTIN (*n* = 3, NT vs 1.0 μM *p = .0395, unpaired *t* test). (**C**) Representative confocal images of myotubes differentiated for 7 d from SYUIQ-5 untreated (NT) or treated (0.4 μM and 1.0 μM) myoblasts and incubated with MitoTracker (red channel) before being fixed. Cells have been immunolabeled with anti-MyHC (green channel) to select differentiated myoblasts and nuclei have been stained with DAPI (blue channel). Objective 20×, scale bar 20 μM. Histogram referred to mitochondria content measured as mean fluorescence intensity signal of MitoTracker (*n* = 47–128, NT vs 0.4 μM ****p < .0001, NT vs 1.0 μM ****p < .0001, 0.4 μM vs 1.0 μM *p = .0423, Kruskal–Wallis Dunn’s multiple comparison test). (**D**) Histogram of MYOSTATIN quantification in supernatant of myotubes differentiated from nontreated myoblasts (NT) and from myoblasts treated with 0.4 μM and 1.0 μM of SYUIQ-5. MYOSTATIN levels are expressed as pg/ml and detected by ELISA assay (*n* = 5, NT vs 1.0 μM *p = .0226, 1-way ANOVA Tukey’s multiple comparison test).

Each experiment was repeated on at least 3 independent cultures and statistical analysis was performed with Prism v. 8.3.0 (GraphPad Software Inc., La Jolla, CA, USA). Statistical significance was determined using a parametric test (1-way ANOVA) for data with normal distribution or a nonparametric test (Kruskal–Wallis) for data without normal distribution. Tukey’s, Dunnett’s, or Dunn’s multiple comparison tests were used, as indicated in the corresponding figure legends. Data are presented as mean ± standard deviation (*SD*). All data were normalized on the levels detected in untreated conditions (NT). Differences were considered significant for *p* < .05 (*p < .05; **p < .01; ***p < .001; **** *p* < .0001). *N* represents the number of cells (Figures 4B and 5C, Supplementary Figure 4) or the biological replicates (Figures 1A and B, 2, 3, 4A, 5A and B; Supplementary Figures 1–3).

## Results

### High Dose of SYUIQ-5 is Toxic for Myoblasts While Adhering Cells are Metabolically Active

Because SYUIQ-5 has never been used on C2C12, we first defined which concentration could represent the best condition to treat these cells without inducing any cell death.

LDH cytotoxic assay revealed that 2 µM treatment is toxic for C2C12 cells. In contrast, 0.4 µM, 0.7 µM, and 1 µM treatments had no toxic effects (Figure 1A). We observed that the amount of LDH released in the supernatant did not change in any conditions (Figure 1A, left panel). In comparison, the intracellular LDH amount significantly decreased after SYUIQ-5 2 µM treatment (Figure 1A, middle panel: SYUIQ-5 NT mean = 1, SYUIQ-5 2 µM mean = 0.2078). LDH amount released in the supernatant normalized on intracellular LDH enzyme confirmed the overall toxicity of SYUIQ-5 2 µM for 72 hours (Figure 1A, right panel: SYUIQ-5 NT mean = 1, SYUIQ-5 2 µM mean = 9.032).

We then performed an Alamar Blue assay on C2C12 cells after acute (2 hours) and chronic (72 hours) SYUIQ-5 treatments to evaluate their viability. We observed no significant differences in the Alamar Blue signal among all the SYUIQ-5 concentrations used (0.4 µM, 0.7 µM, 1.0 µM, and 2.0 µM), at both time points (Figure 1B, left panel). However, since the reduction in protein content after chronic treatments concomitantly with increasing SYUIQ-5 concentration (Figure 1B, right panel), we normalized the results of the Alamar Blue assay on the total protein content and, even in this case, we did not observe any significant differences between the treatments and the control condition (Figure 1B, middle panel). This showed that the remaining adherent cells were metabolically active and viable, an essential feature of senescent cells, revealing that doses of SYUIQ-5 below 2 µM have no toxic effects. Thus, based on this evidence, we decided to proceed further with untreated cells and cells treated with SYUIQ-5 0.4 µM and 1.0 µM, for 72 hours.

### 1 µM of SYUIQ-5 Induces the Expression of the Main Hallmarks of Senescence in C2C12 Cells after 72 hours of Treatment

The peculiarity of senescent cells is that they express a heterogeneous phenotype characterized by several features, mainly depending on cell type, senescent stimulus, and the timing of senescence induction (7,14), making their identification difficult. Thus, to define a cell as senescent is necessary to confirm the coexistence of more features simultaneously.

#### Cell cycle and proliferation arrest

We first investigated if SYUIQ-5 could induce cell cycle and proliferation arrest in myoblasts after 72 hours. BrdU ELISA analysis revealed a dose-dependent reduction in C2C12 proliferation rate, with the significance reached for 1 µM and no significative reduction in cell number (Figure 2A, −22% with 0.4 µM, −56% with 1 µM SYUIQ-5, compared to NT, Supplementary Figure 1A). Furthermore, in accordance with a reduction in cell proliferation, C2C12 cells expressed higher transcript and protein levels of the cyclin-dependent kinase inhibitor (CDKI) 1 (p21), an important negative regulator of cell proliferation, after treatment with 1 µM of SYUIQ-5 compared to NT (Figure 2C and D).

#### Lysosomal content increase

One of the most common features of cell senescence is the increased lysosomal content, which is assessed by measuring the senescence-associated β-galactosidase (SA-βgal) enzymatic activity (15). For a more specific measurement, we detected SA-βgal activity by FACS analysis; an increase in the percentage of SA-βgal positive cells was observed after SYUIQ-5 0.4 µM and 1 µM treatment, that is, 17% and 22%, respectively, as well as an increase in the mean fluorescence intensity of SA-βgal signal (Figure 2B).

#### DNA damage response activation

We then investigated the DDR induced by DNA damage, a typical hallmark of senescence (7). We observed the enhancement of histone H2AX phosphorylation (ƴ-H2AX) by western blotting (Figure 2E left panel; Supplementary Figure 1B), together with a concomitant enrichment of ƴ-H2AX nuclear foci by immunofluorescence analyses, in treated cells compared to NT (Figure 2E, right panel). Additionally, SYUIQ-5 treatment contributed to the enhanced phosphorylation of p53 (Figure 2F; Supplementary Figure 1C). These pieces of evidence suggest that SYUIQ-5 induces senescence as a consequence of DDRs activation.

#### Activation of neither autophagy nor apoptotic pathways

Interestingly, the selected SYUIQ-5 concentrations used for C2C12 treatment did activate neither autophagy nor apoptosis pathways. Indeed, we did not observe any increase in LC3A/B II related to LC3A/B I autophagy protein expression (Figure 3A; Supplementary Figure 2A) or in the activity of apoptotic enzymes, such as CASPASE-3 and CASPASE-7 by using western blotting analysis and a specific enzymatic activity assay (Figure 3B and C). Neither full-length CASPASE-3 and CASPASE-7 diminished after SYUIQ-5 treatment, nor CASPASE-7 cleaved fragment increase after SYUIQ-5 treatment (Figure 3B). These results are corroborated by the observation of increased p21 levels (Figure 2C and D) that are known to protect senescent cells from death by restricting Jun N-terminal kinases (JNK) and caspase signaling under persistent DDR (16).

These data reveal that 1 µM of SYUIQ-5 72 hours in C2C12 cells is sufficient to induce a senescent phenotype without inducing apoptosis or autophagy.

### SYUIQ-5-treated C2C12 Myoblasts Showed Impaired Differentiation, Fusion, and Maturation Potential into Myotubes at Different Time-points

To test whether the senescent phenotype induced in myoblasts could affect their differentiation potential into myotubes, we treated C2C12 cells for senescence and differentiated them for 4 and 7 days by supplementing the medium with 2% horse serum.

Treated myoblasts demonstrated a reduced capability to differentiate into myotubes, as shown by western blotting and immunofluorescence analysis (Figure 4). Western blotting analysis revealed that MyHC protein levels were drastically reduced (about half of the control condition) in SYUIQ-5 myotubes differentiated for 7 days compared to their untreated counterpart (Figure 4A). Immunofluorescence analysis revealed a significant reduction in differentiation potential, fusion, and maturation indexes of myoblasts treated with 0.4 µM and 1 µM of SYUIQ-5 compared to the untreated condition (Figure 4B).

### Myotubes Derived from SYUIQ-5-treated C2C12 Myoblasts Expressed Sarcopenic-like Features

Accumulation of senescent cells is a typical feature of the aged muscle-skeletal system and their presence within the muscle tissue, in vivo, induces the expression of sarcopenic features (17,18). Thus, we analyzed the myotubes differentiated from myoblasts treated with SYUIQ-5 to investigate potential sarcopenic-like characteristics as a consequence of the senescence induced in myoblasts.

#### Induction of a pro-catabolic phenotype and decreased mitochondria content

Western blotting analyses and immunofluorescence experiments performed on myoblasts treated with 1 µM of SYUIQ-5 and differentiated into myotubes revealed significantly increased expression of 2 E3 ubiquitin-ligases, muscle RING-finger protein-1 (MURF1), and ATROGIN-1 (Figure 5A. MURF1 NT = 1 vs 1 µM = 1.83; ATROGIN-1 NT = 1 vs 1 µM = 1.53; Supplementary Figure 4), that are associated with enhanced protein degradation through the ubiquitin-proteasome dependent proteolytic pathway typical of sarcopenic muscles (19). We interestingly observed that the increased levels of these proteins correspond to increased ubiquitination levels (Figure 5B) suggesting an enhanced protein degradation in myotubes differentiated from senescent myoblasts. Furthermore, since the concerns about the specificity of the commercially available antibodies for ATROGIN-1, the experiment has been replicated with another antibody (ATROGIN-1, # AM3141, ECM Biosciences) with comparable results (Supplementary Figure 3).

Another important characteristic of sarcopenic muscles is the impaired mitochondrial function associated, among other causes, with decreased mitochondria content and impaired fusion/fission rate (20). Interestingly, mitotracker analysis revealed a substantial reduction in mitochondria content in myotubes derived from 0.4 µM and 1 µM SYUIQ-5-treated myoblasts compared to NT (Figure 5C. Reduction of almost 40% and 50% in 0.4 µM and 1 µM condition respectively compared to NT).

#### Increased MYOSTATIN release

The well-known endocrine function of skeletal muscles is explicated via releasing several mediators (ie, myokines) that act through autocrine and paracrine fashion (21). Senescent cells are commonly characterized by a marked SASP and their secretome is supposed to differ from that of healthy cells. Thus, we hypothesized that myotubes derived from SYUIQ-5 treated myoblasts could express a different myokines profile, compared to the control condition, and, among them, a different expression level of MYOSTATIN, a mediator relevant in protein metabolism in muscle and associated with the aged muscle phenotype (22). Myotubes derived from 1 µM SYUIQ-5 treated myoblasts released significantly higher levels of MYOSTATIN than the other conditions and, notably, 50% higher than the untreated control (Figure 5D).

These data suggest that myotubes differentiated from 1 µM SYUIQ-5 treated myoblasts are characterized by features typical of sarcopenic myotubes and release increased levels of MYOSTATIN that could affect skeletal muscle cell growth, thus contributing to sarcopenia.

## Discussion

We demonstrated for the first time that SYUIQ-5 can induce senescence in murine cultured myoblasts, defining the best SYUIQ-5 treatment condition to generate a new C2C12 cells-based in vitro model of skeletal muscle senescence.

Muscle impairment is a typical feature in aging that usually could evolve into sarcopenia, a pathological condition characterized by the decline in skeletal muscle mass, strength, and function (23), associated with various adverse health outcomes in geriatric patients. Although the relevant clinical impact of this condition, the specific mechanisms underlying the onset and development of muscle impairment associated with aging are still mostly unknown.

Accumulation of senescent cells during aging affects tissues, particularly the skeletal muscle, and they are supposed to contribute to the expression of a sarcopenic phenotype, usually associated with bone impairment and frailty (8,24). The susceptibility of skeletal muscle cells to senescence with advancing age is not evident. The mechanism leading to muscle impairment and sarcopenia, associated with the presence of senescent cells, is even less known. However, a recent study demonstrated that cell senescence is a core feature of skeletal muscle aging and a potential contributor to sarcopenia, showing that different types of skeletal muscle cells are susceptible to senescence with aging (8).

A senescent state could be caused by the activation of several different mechanisms. Telomeres length and functions are closely associated, among several biological processes in which they are involved, with replicative senescence and cell cycle arrest (25) and, to the best of our knowledge, there are no in vitro models of senescent myoblasts obtained through the treatment with a molecule able to induce telomeres damage. Furthermore, this approach is considered less aggressive and the amount and type of phenotypic changes induced are more similar to the “physiological” senescence compared to other types of senescence-inducing stimuli, such as H_2_O_2_, that, other than being highly stressful, act on a single mechanism. Thus, as a senescent stimulus, we decided to use SYUIQ-5, a quindoline derivative able to inhibit telomerase action reported to induce senescence in some cell lines (10,26).

C2C12 mouse myoblasts are considered a relevant model for studies related to muscle functions and their ability to differentiate into myotubes under low-serum conditions.

Thus, we used SYUIQ-5 on proliferating C2C12 cells to mimic the replicative senescence brought by telomeres erosion by using concentrations below 2 µM to avoid autophagy induction (27). Indeed, it has been shown that C2C12 cells, differently from primary isolated myoblasts and more similar to satellite stem cells, retain telomerase activity during several passages in vitro (28); consequently, replicative senescence in C2C12 cells caused by telomeres shortening could be hard to obtain. With SYUIQ-5, we can induce premature senescence in myoblasts within a relatively short time throughout telomerase inhibition that could mimic the replicative senescence that usually can be obtained longer.

A strength of this model is that starting from senescent SYUIQ-5 myoblasts, we were able to obtain, after a proper differentiation, myotubes expressing relevant age-related features, such as sarcopenic muscles, in a fast, simple, and highly reproducible way, thus providing a novel tool for the study of muscle aging.

### Hallmarks of senescence characterizing SYUIQ-5 treated C2C12 cells

The heterogeneous phenotype of senescent cells makes difficult their univocal identification and complicates the studies of pathological consequences related to their presence. To define a cell as senescent, it is necessary to simultaneously analyze and identify more senescent markers (7,14). Going deeply through all the possible senescent-associated pathways that could be activated after SYUIQ-5 treatment, we showed that the resulting phenotype, induced in C2C12 myoblast, recapitulates several features of the senescent cells. A common hallmark in cell senescence is represented by the cell-cycle arrest associated with the increased expression of CDKIs (29). In our model, this feature is related to the activation of the p21 pathway. Furthermore, the enhanced phosphorylation of p53, suggestive of p53 transactivation, as a part of a more generic DDR (30), could be responsible for p21 activation. However, because p21 activation could also be enriched in a p53-independent manner (31), it becomes necessary to further detail the direct link between p21 and p53. Considering also that p53 activation could be induced or downregulated according to senescent status (ie, early or late senescence (32)), it could be interesting to explore it to improve our model. In support of the idea that SYUIQ-5 induces DDR, which in turn remains unresolved, we detected the accumulation of nuclear foci enriched in phosphorylated H2AX histone, which are aberrant structures arising at dysfunctional telomeres, typical of replicative senescence (33). These observations corroborate the hypothesis that SYUIQ-5 shortens telomeric length that activates DDR, leading to cell-cycle arrest through p53 and p21 signaling pathways, thus responsible for senescence induction in C2C12 cells.

Another important senescence-associated feature induced by SYUIQ-5 treatment is the enhanced SA-βgal enzyme activity. This is the most widely used marker of senescence and it is usually associated with increased lysosomal accumulation (15). Finally, a further confirmation of the senescent phenotype induced in our model came from the activation of neither autophagic nor apoptotic pathways, which are usually inhibited in senescent cells (3).

### Sarcopenic-like myotubes resulting from senescent SYUIQ-5-treated myoblasts

New recent hypotheses suggest cellular senescence as a potential contributor to sarcopenia by linking the loss of muscle regenerative potential observed in age-related sarcopenia to senescence-associated alterations in muscle stem cells (8,34). Furthermore, an interesting study shows that the trans-plantation of senescent muscle cells into healthy muscle fibers induced increased senescent markers in the muscles and sarcopenic features in muscle fibers (18). However, the specific mechanisms that lead to the differentiation in sarcopenic myotubes of senescent myoblasts are not well known and this may be related to the lack of adequate experimental models. Our in vitro model of muscle senescence could support these investigations. Indeed, we showed that the SYUIQ-5-treated myoblasts expressed low amounts of MyHC protein and displayed impaired differentiation potential with a low rate of fusion events. Mitochondria dysfunction and impaired proteostasis are important contributors to the complex etiology of sarcopenia (35). We demonstrated that the obtained myotubes were less mature than the untreated counterpart, with fewer nuclei per cell. Furthermore, they had a reduced mitochondria content, suggesting impairment in mitochondria dynamics, biogenesis, or mitophagy may lead to endoplasmic reticulum (ER) stress. This latter point leaves open questions about mitochondria and ER alteration typical of sarcopenic muscles that could be deeply explored with our model of aged muscle cells. We also showed that the obtained myotubes expressed high levels of MURF1 and ATROGIN-1 proteins, resulting in increased levels of total UBIQUITIN protein. These latter are key muscle-specific ubiquitin ligases, pivotal regulators of protein turnover, and, therefore, central contributors to muscle protein degradation and functional impairment strongly associated with muscle atrophy (36,37). Moreover, the sarcopenic phenotype is characterized by the change in the secretory profile with the release of factors, acting autocrine or paracrine (38), that further enhance the pro-catabolic phenotype, such as MYOSTATIN that we found highly expressed in our model. Interestingly, the increased MYOSTATIN released levels have been already observed in multiple population that could mimic our senescent myoblasts model as replicative senescence, thus reinforcing our observations (39,40). MYOSTATIN is a negative regulator of myogenesis and it has been shown to contribute to the induction of ATROGIN-1 and MURF1 proteins (41), thus potentially activating a vicious cycle that pushes further toward muscle wasting, characterizing the aging phenotype (42). These observations suggest that accumulation of senescent cells leading to sarcopenic phenotype may also affect the released myokines’ profile from impaired muscles that could negatively affect various cells and organs, thus contributing to a general impairment of healthy status typical of aging phenotype. As is now well known, sarcopenia is often associated with bone frailty during aging (43). Besides its adverse effects on muscle tissues, MYOSTATIN may also affect bone status by acting as a negative regulator of bone formation and metabolism (44). Furthermore, it has been shown that plasma abundance of MYOSTATIN negatively correlated with bone mineral density (BMD) (45). MYOSTATIN dysregulation could be a main determinant for the coexistence of bone and muscle impairment observed in older adults.

A still unanswered question is: what type of myoblasts retain the ability to differentiate into sarcopenic-like myotubes? Or, in other words, are the senescent myoblasts differentiating into sarcopenic-like myotubes, or do the senescent myoblasts determine an environment that induces the neighboring cells to differentiate into dysfunctional myotubes? The answer to this intriguing question requires deeper investigations, which could improve the knowledge about the pathophysiological mechanisms involved in skeletal muscle aging. Furthermore, this knowledge would better define the utility of targeting senescent cells as a novel strategic therapy to ameliorate aging. Indeed, it has already been demonstrated that the use of senolytics (ie, drugs able to eliminate senescent cells) or senomorphic agents (ie, compounds able to modulate the adverse effects associated with the expression of SASP) could improve specific impairment associated with the presence of senescent cells (46,47). Furthermore, in a view specifically addressed to the muscle-skeletal apparatus, it has been shown that a systemic deletion of senescent cells improves physical function and slowdowns the progression of sarcopenia (48). Additional investigations are needed to introduce tools to univocally identify the senescent cells within the skeletal muscle in order to find out new specific biomarkers useful to monitor novel therapies (eg, those based on the use of senolytic or senomorphic drugs) (8,49–51). In this context, our in vitro model, by recapitulating relevant pathophysiological mechanisms proper of the aged skeletal muscle, may result in a useful tool in aging research.

## Supplementary Material

glae022_suppl_Supplementary_Figures_S1-S4
