# Supplementary material for: Murine Myoblasts Exposed to SYUIQ-5 Acquire Senescence Phenotype and Differentiate into Sarcopenic-Like Myotubes, an In Vitro Study
Source: J Gerontol A Biol Sci Med Sci. 2024 Jan 24;79(4):glae022. doi: 10.1093/gerona/glae022 (PMC10924451; doi:10.1093/gerona/glae022)
Supplement: glae022_suppl_Supplementary_Figures_S1-S4 [file glae022_suppl_supplementary_figures_s1-s4.pdf]

SUPPLEMENTAL MATERIALS

Supplemental figures: 4 figures, named Supplementary Figure1, Supplementary Figure 2, Supplementary Figure 3 and Supplementary Figure 4

Supplemental figure captions: 1 page with 4 supplemental figure legends

Supplementary Figure 1 (A) Histogram representing the number of cells/ml after SYUIQ-5 treatments (0.4 µM, 1.0 µM) compared to untreated condition (NT). Means: NT= 1.000, 0.4 µM= 0.9372, 1.0 µM= 0.7760. n= 4, one-way ANOVA Kruskal Wallis Dunn's multiple comparison test, ns. **(B)** Quantification of total H2AX protein levels (left histogram) and of phosphorylated H2AX protein levels (γ-H2AX, right histogram) normalized on VINCULIN (n = 5, ns, Kruskal-Wallis Dunn's multiple comparison test). **(C)** Quantification of total p53 protein levels (left histogram) and of phosphorylated p53 protein levels (right histogram) normalized on VINCULIN (n = 6, NT vs 1.0 µM * p = 0.0125, Kruskal-Wallis Dunn's multiple comparison test).

Supplementary Figure 2 (A) Histograms relative to LC3 A/B I protein levels quantification (left histogram) and to LC3 A/B II protein levels quantification (right histogram) normalized on VINCULIN (n = 3, ns, Kruskal-Wallis Dunn's multiple comparison test).

Supplementary Figure 3 Western blotting images of ATROGIN-1 protein expressed in 3 different experiments (exp1, exp2 and exp3) of myotubes lysates differentiated for 7 days from SYUIQ-5 untreated (NT) or treated (1.0 µM) myoblasts and relative histogram of protein levels quantification normalized on VINCULIN (ATROGIN-1 histogram: n = 3, NT vs 1.0 µM * p = 0.0217, unpaired t-test). Anti-ATROGIN-1 cod. AM3141, ECM biosciences,Versailles, KY, United States).

Supplementary Figure 4 Histograms relative to the mean fluorescence intensity of ATROGIN-1 (left panel) and MURF1 (right panel) specific of myotubes (selected region of interest based on MyHC channel) differentiated for 7 days from SYUIQ-5 untreated (NT) or treated (1.0 µM) myoblasts. Representative confocal images of myotubes immunolabeled with anti-MyHC (green channel), ATROGIN-1 (red channel) and MURF1 (blue channel). Objective 20X, scale bar 100 µm. Statistical analyses: unpaired t-test, **** p < 0.0001, n=30).

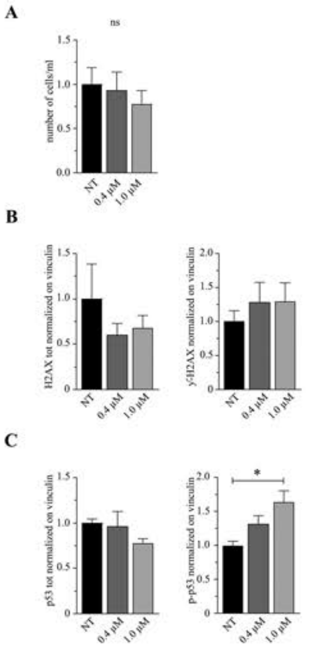

Supplementary Figure 1

A

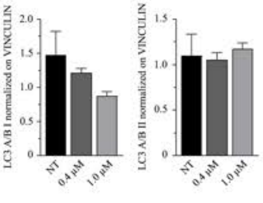

Supplementary Figure 2

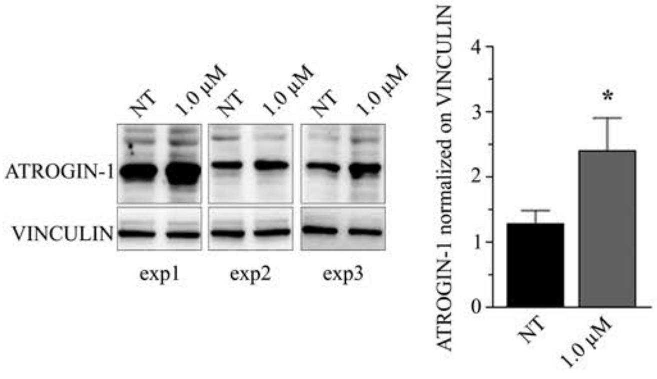

Supplementary Figure 3

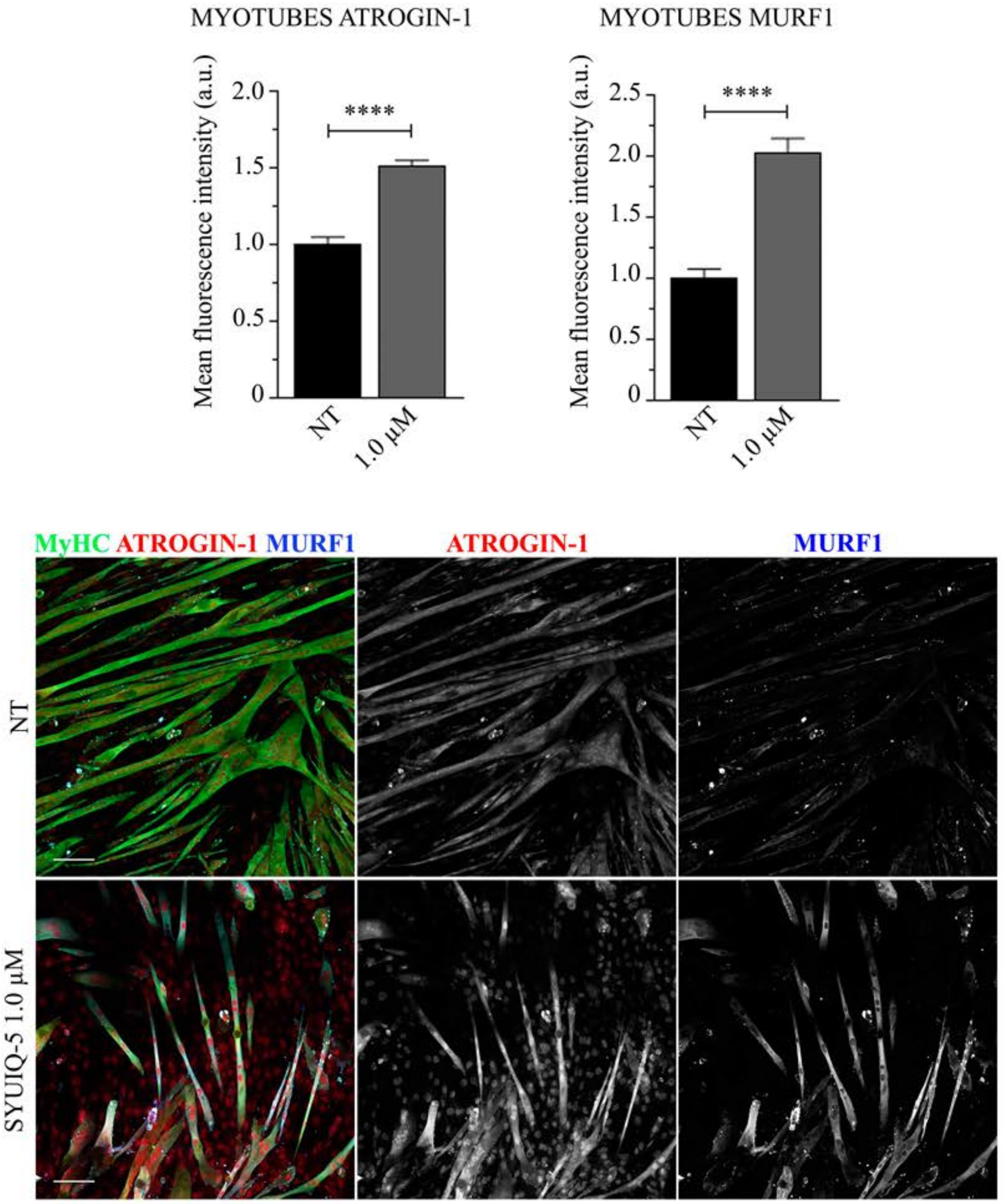

Supplementary Figure 4
